# New Insights into the Biological and Pharmaceutical Properties of Royal Jelly

**DOI:** 10.3390/ijms21020382

**Published:** 2020-01-08

**Authors:** Saboor Ahmad, Maria Graça Campos, Filippo Fratini, Solomon Zewdu Altaye, Jianke Li

**Affiliations:** 1 Key Laboratory of Pollinating Insect Biology, Ministry of Agriculture, Institute of Apicultural Research, Chinese Academy of Agricultural Sciences, Beijing 100081, China; 2018Y90100172@caas.cn (S.A.); 2017Y90100103@caas.cn (S.Z.A.); 2 Coimbra Chemistry Centre, (CQC, FCT Unit 313), Faculty of Sciences Technology, University of Coimbra, Rua Larga, 3004-535 Coimbra, Portugal; mgcampos@ff.uc.pt; 3 Observatory of Drug-Herb Interactions, Sciences Health Campus, Faculty of Pharmacy, University of Coimbra, Azinhaga de Santa Comba, 3000-548 Coimbra, Portugal; 4 Department of Veterinary Sciences, University of Pisa, Viale delle Piagge 2, 56124 Pisa, Italy; filippo.fratini@unipi.it; 5 Interdepartmental Research Center “Nutraceuticals and Food for Health”, University of Pisa, Via del Borghetto 80, 56124 Pisa, Italy

**Keywords:** royal jelly, bioactive compounds, functional properties, proteins, fatty acids, phenolics

## Abstract

Royal jelly (RJ) is a yellowish-white and acidic secretion of hypopharyngeal and mandibular glands of nurse bees used to feed young worker larvae during the first three days and the entire life of queen bees. RJ is one of the most appreciated and valued natural product which has been mainly used in traditional medicines, health foods, and cosmetics for a long time in different parts of the world. It is also the most studied bee product, aimed at unravelling its bioactivities, such as antimicrobial, antioxidant, anti-aging, immunomodulatory, and general tonic action against laboratory animals, microbial organisms, farm animals, and clinical trials. It is commonly used to supplement various diseases, including cancer, diabetes, cardiovascular, and Alzheimer’s disease. Here, we highlight the recent research advances on the main bioactive compounds of RJ, such as proteins, peptides, fatty acids, and phenolics, for a comprehensive understanding of the biochemistry, biological, and pharmaceutical responses to human health promotion and life benefits. This is potentially important to gain novel insight into the biological and pharmaceutical properties of RJ.

## 1. Introduction

Royal jelly (RJ) is known as a “superfood” which is produced by nurse bees to feed young worker larvae and queen bees [1,2]. The major components of RJ are (60–70% *w*/*w*) water, (9–18% *w*/*w*) proteins, (7–18% *w*/*w*) sugars, and (3–8% *w*/*w*) lipids [3,4]. RJ also contains minor components, such as minerals (Fe, Na, Ca, K, Zn, Mg, Mn, and Cu), amino acids (eight essential amino acids Val, Leu, Ile, Thr, Met, Phe, Lys, and Trp), vitamins (A, B complex, C, and E), enzymes, hormones, polyphenols, nucleotides, and minor heterocyclic compounds [3,5,6]. RJ is an active research domain because it is essential for larval development and queen reproduction in honeybee colonies through the metabolism of sugars, lipids, and proteins [7,8]. Thereby, the larger body size, longer lifespan, and fertility of queens compared to worker bees are potentially correlated to the special diet of RJ [9].

RJ has been produced in large scale for commercial purposes to date, and its market value is significantly higher than other bee products, such as honey or pollen, thus, it is a major income source for beekeepers [4,10]. Beekeepers have made great efforts to develop the technique to improve RJ production and to select for high-producing strains of honeybees. For instance, the increase in the production of RJ in China over the last 40 years has been achieved by the development of genetic selection of high RJ-producing bees (RJBs) from Italian bees [11,12,13], and the development and implementation of production techniques to increase and optimize RJ production [14,15,16]. At present RJBs have the potential to produce more than 10 kg RJ/colony/year, which is 10 times more than for non-selected Italian bees [15,16,17,18]. Notably, China is the largest producer and exporter of RJ around the world, producing more than 4000 tons annually, with more than $2.5 billion market, which is 90% of the total RJ production globally and mostly exported to Japan, Europe, and the United States [12,19].

**Figure 1:**
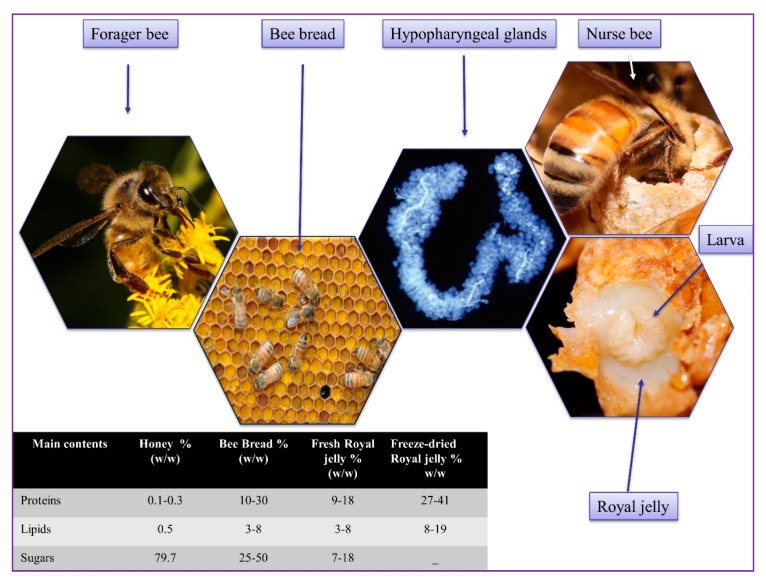
Forager bees transport pollens in their hind leg corbiculae to which they add nectar to form pollen pellets. Forager bees deposit and pack the pollen pellets into cell surrounding the brood area and forming bee bread. Nurse bees develop enlarged food glands and produce RJ by consuming honey and bee bread (Photos taken by Prof. Dr. Jianke Li).

**Figure 2:**
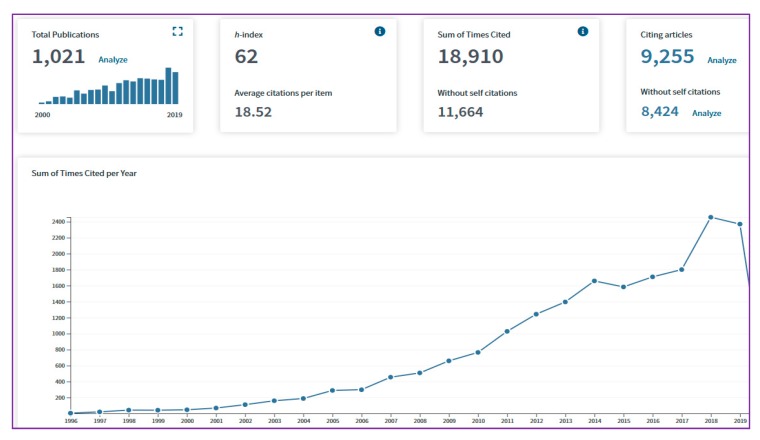
Numbers of publications on RJ that appear from international journals are increasing every year (data from the core collection of the Web of Science).

The health-promoting benefits and pharmaceutical properties of RJ from animal models to humans have been widely investigated. RJ is a nutritional modification of honey and bee bread (Figure 1), and it is commercially available on a large scale as health food and cosmetics in Asia, especially in China and Japan [9,10]. Moreover, RJ is used to explore further applications as a drug and traditional consumption as “remedies” for humans and animals [20]. To date, the importance of RJ has attracted attention around the world, which is evidenced by the growth in the number of publications and citations in the core collection of the Web of Science (Figure 2). Recently, the origin and function of RJ, such as major royal jelly proteins (MRJPs) for the development of the larvae [21], antimicrobial properties [9], medicinal value [20,22], proteins and peptides [23], the potential applications for cancer treatment [24], and health aging and longevity [25] have been reported. To better understand the biochemistry, biological, and pharmaceutical response to health and life benefits of RJ, we update the knowledge from the research advances of the biological activities and pharmaceutical applications of RJ and its bioactive ingredients that are associated with farm animals, micro-organisms, laboratory animals, insects, and clinical trials in humans. Here, our major focus is on the bioeffects of RJ, such as antimicrobial, antioxidant, anti-inflammatory, wound healing, anti-aging, immunomodulatory, anti-cancer, anti-diabetic, anti-hyperlipidemic, anti-hypertension, hepato-renal protective, neuroprotective, estrogenic, and fertility effects. This evidence is a potentially valuable resource for further studies of the health potential properties of RJ for both humans and honeybees.

## 2. Bioactive Substances

**Figure 3:**
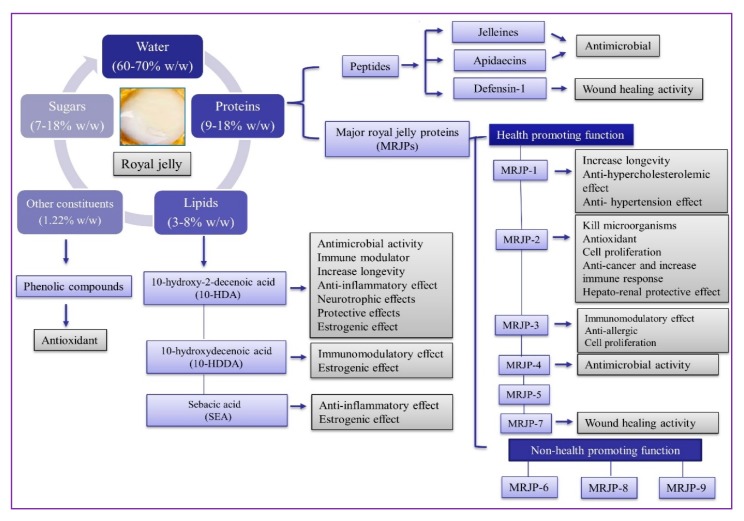
A schematic representation of the main biological substances in RJ and their functional activities. For detailed information refer to Table 1.

RJ is a rich source of nutrients and bioactive compounds with the potential to play a vital part in their biological activities and pharmaceutical applications [26]. It has been confirmed that proteins, peptides, lipids, phenolics, and flavonoids are the main bioactive compounds responsible for the various pharmaceutical properties of RJ (Figure 3). The natural variation of bioactive compounds depend upon the biodiversity of flora species present in the different ecosystem [26].

### 2.1. Proteins and Peptides

The investigation of novel proteins in RJ has been a long-term perusal for biochemical experts and apicultural biologists. Proteins are the most abundant components of RJ, accounting for more than 50% of the dry weight and MRJPs are the most important components constituting 80%–90% of the total protein content [21,27]. Others are glucose oxidase [27], α-glucosidase, and α-amylase [28]. The MRJPs share a common developmental genesis with the yellow protein family [29]. The YELLOW/MRJPs are named according to their molecular weight or simply numbered by the order in which they are discovered. So far, MRJPs (1–9) are well-described with molecular mass 49–87 kDa, which are encoded by nine different genes [23,30]. MRJP-1 is a weak acidic glycoprotein, accounting for 48% of water-soluble RJ proteins and the secondary structure consists of 9.6% α-helices, 38.3% β-sheets, and 20% β-turns [6,31]. Particularly, MRJP-1 occurs as a monomer (mono MRJP-1) or as an oligomer known as apisin by polymerization with apisimin [31]. Apisin could be used to determine the quality of RJ [32]. MRJP-2 and MRJP-3 produced by Chinese bees (*Apis cerana cerana*) are less polymorphic compared to European bees (*Apis mellifera ligustica*) and Africanized bees (*Apis mellifera scutellate*) [33,34], and MRJP-4 was first time confirmed by two-dimensional gel electrophoresis (2-DE) analysis during the comparison in the RJ of Africanized and European bees [34]. The important feature of MRJP-5 is a wide repeated region located between amino acid residues 367 and 540 [33]. The most significant post-translational modification of the MRJPs is methylation which triggers polymorphism of MRJP 1–5 in the RJ [35]. MRJPs 6–9 are recognized in RJ through proteomic analysis [36,37]. Furthermore, 1-peroxiredoin and 1-glutathione S-transferase are identified in RJ [11]. RJ also contains a calcium-binding protein, known as regucalcin, and a lipid-binding protein, such as apolipophorin-III [38]. Phosphorylated icarapin (venom protein-II) and apolipophorin-III-like proteins are identified in RJ may promote the strength of immunity [39]. There are 53 N-glycosylation sites residing on 25 *N*-glycosylated proteins in RJ. Most of the glycosylated proteins are associated with metabolic activities and health benefits [12,28].

RJ is rich in amino acids, including lysine, proline, cysteine, aspartic acid, valine, glutamic acid, serine, glycine, cysteine, threonine, alanine, tyrosine, phenylalanine, hydroxyproline, leucine, isoleucine, and glutamine [22,40]. These high amounts of amino acids in the MRJPs family is essential for developing of both queen bees and larvae. Amino acids, such as arginine, leucine, isoleucine, histidine, lysine, threonine, tryptophan, methionine, valine, and phenylalanine, are most commonly present in MRJPs, with MRJP-1 to 9 contains 48%, 47%, 39.3%, 44.5%, 51.4%, 42%, 48.3%, 49.5%, and 47.3% of these amino acids, respectively. The major amino acids in MRJP-1, MRJP-2, and MRJP-4 are valine and leucine. MRJP-3 is rich with arginine and lysine while the prominent amino acids in MRJP-5 is methionine and arginine. Furthermore, leucine is the major amino acid in MRJP-(6–8) and isoleucine is the rich one in MRJP-9 [41,42]. The MRJPs provide nutritive components such as essential amino acids to RJ.

Similar to proteins, peptides represent a specific sequence of amino acids in RJ that has biological activity with health effects and potential applications. They can be identified by proteomics, such as jelleines-I, jelleines-II, jelleines-III, jelleines-IV, and jelleines, are identical to the C-terminal of the MRJP-1 [43]. Moreover, RJ also contains peptides including apidaecin, defensin, hymenoptaecin, jelleine-II, jelleine-II (pT), and jelleine-II (pS) [39,44]. Phosphorylated jelleine-I (pS), jelleine-II (pS), and jelleine-IV (pS) are found in *Apis cerana* RJ while jelleine-II (pT) and jelleine-IV (pT) in *Apis mellifera* RJ [39].

### 2.2. Lipids and Fatty Acids

A distinctive feature of RJ is associated with its lipids and fatty acids content. The lipids are 80%–85% of free fatty acids with few being esterified. This fraction also includes 4–10% phenolic compounds, 5–6% waxes, 3–4% steroids, and 0.4–0.8% phospholipids. RJ contains a medium-chain fatty acids, normally 8–12 carbon atoms, some hydroxylated in terminal or internal position, as mono-hydroxyl fatty acids or dicarboxylic acids, and saturated or unsaturated at the 2-position [45]. About 80–90% fatty acids have a different structure such as 10-hydroxy-2-decenoic acid (10-HDA), 10-hydroxydecenoic acid (10-HDDA), and sebacic acid (SEA). This fraction consists of 32% *trans*-10-HDA, 22% 10-HDDA, 24% gluconic acid, 5% dicarboxylic acids, and some other acids [46]. In addition, fatty acids, such as 8-hydroxy octanoic acid (8-HOC), 3,10-dihydroxydecanedioic acid (3,10-HDecDA), 9-hydroxy-2-decenoic acid (9-HDA), 1,10-decanedioic acid (DecDA), 3-hydroxydecanoic acid (3-HHDA), and 2-decene-1,10-dioic acid (2-DecDA), can also found in RJ [47]. Among all lipids and fatty acids, 10-HDA is a stable compound representing 3.5% of freeze-dried RJ which is considered an international standard for quality [5,46,48,49]. In the lipid fraction sterols should be included, even if they are only in trace amounts. For instance, 24-methylene cholesterol (24-MET) contribute with 49–58% for total sterols in RJ. Other similar compounds include β-sitosterol (19–24%), isofucosterol (9–16%), campesterol (67%), and desmosterol (0.5–4.5%) [3].

### 2.3. Other Constituents

RJ contains some other bioactive compounds, such as 23.3 (µg/mg) of phenolics and 1.28 (µg/mg) of total flavonoids [20,49,50]. The phenolic compounds comprise phenol and carboxylic groups [51]. From flavonoid compounds, various structures could be distinguished, such as flavones (apigenin and its glycosides, luteolin, chrysin, and acacetin), flavanones (naringenin, hesperetin, and isosakuranetin), flavonols (kaempferol and isorhamnetin glycosides), and isoflavonoids (genistein and formononetin). Coumestrol is an isoflavonoid phytoalexin that can also be found in RJ [52]. Furthermore, flavonoids are mostly present in the form of glycosides, and the aglycones are linked by glycosidic bonds to the osidic group [53]. Another unique compound of RJ is adenosine N1-oxide, which is an oxidized product of adenosine at the N1 position of adenine base moiety [54,55]. Adenosine monophosphate (AMP) and adenosine itself are important biomolecules with physiological effects [56,57,58]. Acetylcholine can also be found with a mean concentration of 1 mg/g dry weight [59]. Hormones, gonadotropins, pantothenic acid, testosterone, estradiol, progesterone, and prolactin also were identified in RJ [60,61,62,63,64].

## 3. Functional Properties of RJ

The biological functions of RJ and its application (Table 1) are investigated in vivo and in vitro experimental models, such as laboratory animals (rabbits, mice, rats, and hamsters), microbial organisms (bacteria, fungi, viruses, and nematodes), farm animals (ewes and buffalos), and clinical trials (humans disease treatment), to provides the basis for further developments of its pharmaceutical effects. The biological activities of the RJ are variable and have been correlated to the content of their active ingredients [65].

### 3.1. Biological Activity of RJ

RJ has health benefits effects for both humans and honeybees. It is a natural antibiotic and plays an efficient role in developing the larval stages in blood cells and maintains its ovulatory characteristics during the whole life span. Moreover, RJ has antioxidants with the potential of reducing the risk of cancer, high blood pressure, diabetes, and cardiovascular diseases [94,110,111]. RJ also affects the morphological characters, growth, learning, size, and shape variations in various creatures, such as honeybees, mice, and humans [112].

#### 3.1.1. Antimicrobial Activity

RJ demonstrates strong antimicrobial properties against different pathogens [39,66,67,70], due to the existence of special proteins and peptides [9,43], and the presence of the 10-HDA [9,113]. Moreover, RJ could fight against periodontopathic bacteria, such as *Aggregatibacter actinomycetemcomitans*, *Prevotella intermedia*, *Fusobacterium mucleatum*, and *Porphyromonas gingivalis* [67]. MRJPs (2–5 and 7) reveal antibacterial activity against Gram-negative *E. coli* [114]. Jellenie I, II, III, and IV are important antibacterial peptides in RJ. Although the difference between jellenie (I–IV) is minor, with only one residue difference in the sequence, this slight difference has a significant impact on their antibacterial activities. Jelleine I–III could inhibit both Gram-positive and Gram-negative bacteria whereas Jelleine-IV doesn’t [43]. Antibacterial peptides are positively charged due to the existence of lysine, arginine, and histidine residues that allow them to interact with anionic phospholipids of the cell membrane and collapse it [115]. Royalisin has three intramolecular disulfide bonds between cysteine residues and shows strong antibacterial activity against different types of Gram-positive and Gram-negative bacteria [70]. In addition, native jelleines could inhibit Gram-positive bacteria (*Bacillus subtilis*, *Staphylococcus aureus*, *Paenibacillus larvae*) and Gram-negative bacteria (*Escherichia coli*, *Pseudomonas aeruginosa*). Furthermore, the phosphorylated jelleines (Jelleine-II (pT) and Jelleine-II (pS) could fight against *E. coli* and *B. subtilis*, *P. larvae*, and *E. coli* [39]. MRJPs 2 and 4 act as antimicrobial agents and have a wide range of activity against bacteria (Gram-positive and Gram-negative), fungi, and yeasts. Recombinant MRJP-2 and MRJP-4 could kill microorganisms by attaching to the cell wall of fungi, yeast, and bacteria that damage the structure of the cell wall [66,68]. RJ aqueous fraction has reported a strong inhibition of the growth of *Fusarium* species [73]. RJ has also exhibited antifungal properties against *Syncephalastrum racemosum*, *Aspergillus fumigants*, and *A. niger* [72]. Royalisin also indicates an anti-fungal response against necrotrophic fungus, such as *Botrytis cinerea* [69]. The native jelleine-ll protein presents an inhibitory effect on *Candida albicans* [39,43]. Moreover, 10H∆DA has antifungal potential in inhibiting the growth rate of *Neurospora sitophila* [116]. RJ is effective against *C. albicans* and as an alternative agent to fight this yeast [117]. Fatty acids such as 3,10-HDA, 11S, 10-HDA and 10-acetooxy-2-DEA could strongly inhibit the growth of yeasts, such as *C. tropicalis*, *C. albicans*, and *C. glabrata* [71]. Moreover, RJ could fight against herpes 2 virus, influenza virus, heart virus coxsackie B3, herpes simplex virus type 1 (HSV-1), and certain rhabdoviruses [118,119].

#### 3.1.2. Antioxidant Activity

The antioxidant activity of RJ could be explored as the prevention and treatment of various chronic and degenerative diseases. In the diet of Sprague–Dawley rats fed with contaminated fumonisin (FB) (200 mg/kg) and RJ (150 mg/kg) for three weeks, RJ attenuates the harmful effect of FB via improving glutathione peroxidase formation and reducing the effects of lipid peroxidation and free radical generation [120]. RJ could also recover from cadmium-induced genotoxicity and oxidative stress in mice, which improves the antioxidant status via glutathione (GSH) and reduces malondialdehyde (MDA) production [121]. After rats exposed to cisplatin and carbon tetrachloride, RJ administration could resist against oxidative stress in liver and renal tissues, which is achieved by decreasing MDA production and increasing the concentration of cellular antioxidant enzymes, such as superoxide dismutase (SOD), catalase (CAT), glutathione reductase (GR), and glutathione peroxidase (GPx) [122]. In radiation-induced lung and liver damage of Sprague–Dawley rats, pre- and post-administration of RJ are effective in reducing oxidative stress and increasing antioxidant properties [74]. The antioxidant response of enzyme-treated RJ (ERJ) is confirmed by the reduction of nitric oxide (NO) and intracellular reactive oxidative species, and increased the effect of the antioxidant glutathione and antioxidant SOD levels. Moreover, ERJ has the potential as an oxidative agent to be used for human, as well as animal, diets [123]. Similarly, MRJP-2 has potential action as an antioxidant to protect mammalian and insect cells via decreasing the levels of caspase-3 activity and oxidative stress-induced cell apoptosis followed by increase cell viability [68]. Hydroxyl radicals and hydrogen-peroxide scavenging activity were verified with 29 antioxidant peptides isolated from RJ hydrolysate, in which 12 small peptides having 2–4 residues (Ala-Lys, Phe-Arg, Ile-Arg, Lys-Phe, Lys-Leu, Lys-Tyr, Arg-Tyr, Tyr-Asp, Tyr-Tyr, Leu-Asn-Arg, and Lys-Asn-Tyr-Pro) having the strongest activity. Moreover, three dipeptides (Lys-Tyr, Arg-Tyr, and Tyr-Tyr) in RJ indicate strong scavenging activity due to a donation of the hydrogen atom from their phenolic hydroxyl group [124].

#### 3.1.3. Wound Healing Activity

Wound healing is an important health issue and a wide range of in vivo and in vitro studies indicate that RJ seems play a significant role [125,126]. The development of atopic dermatitis-like skin lesions in picryl chloride treated NC/Nga mouse is suppressed after treatment with RJ. This is achieved by the down-regulating protein of antigen-specific interferon-gamma (IFN-ϒ) production and up-regulation of NO synthase [127]. The application of dose-dependent RJ improves the healing effect of severe oral mucositis in hamsters induced by chemotherapy drug 5-fluorouracil [128]. Oral treatment with RJ could increase the wound healing process in diabetic mice [125]. Moreover, RJ in 5 µg/mL concentration promotes the fibroblasts migration in human beings by altering the level of different lipids and enhance the level of sphingolipids that promote wound healing [125]. Moreover, an RJ dressing is a good way of treating diabetic foot ulcer patients along with other standard methods. Furthermore, this method creates vasodilation effects around the wound which could dilate blood vessels to increase blood flow and prevent the wound from infection by other microbial organisms [129]. In addition, RJ promotes the wound healing response to control dermal infection induced by methicillin-resistant *S. aureus* (MRSA) [126]. Water-soluble proteins of RJ and its fractions induce proliferative and migratory effects on a human epidermal keratinocyte in a scratch wound model. A protein fraction, mainly containing MRJP-2,3,7 have the potential to influence wound healing bioactivity by stimulating keratinocyte growth and migration suggests that these proteins promote the development of new wound healing medication [130]. The defensin-1 peptide in RJ contributes to skin regeneration and cutaneous wound closure by increasing matrix metalloproteinase-9 secretion and keratinocyte migration [131].

#### 3.1.4. Immunomodulatory Activity

Immunomodulatory response plays a significant role in allergy, cancer, and inflammation by activation of antibody formation or inhibition of white blood cell activities [132]. The first human study in systemic lupus erythematosus (SLE) in children reveals that the effect of RJ treatment in SLE indicates a significant improvement after three months of administration [133]. The anti-allergic factors of RJ inhibit interleukin-4 (IL-4) production which is induced by anti-CD3 activated spleen cells derived from ovalbumin (OVA)/alum-immunized mice. MRJP-3 (70 kDa) glycoprotein inhibits IL-4, IL-2, and IFN-ϒ production by T cells associated with the suppression of cell proliferation. Intra-peritoneal MRJP-3 administration indicates inhibition in immune serum level of anti-OVA IgG1 and IgE in OVA/alum-induced allergic mice while heat-treated soluble MRJP-3 administration decreases antigenicity and maintains its inhibitory effect on antibody response to ovalbumin. Both in vivo and in vitro studies demonstrate that MJRP-3 has strong immunomodulatory activities [84]. Moreover, the lower concentration of water extract of RJ and 3,10-HDA activate the T-cell proliferation by triggering concanavalin A (Con-A) and enhances the production of IL-2 while a higher concentration of water extract of RJ, dry powder of RJ, and trans-10-HDA inhibit T-cell proliferation by decreasing IL-2 and NO production. Water extract from RJ possesses the complexity of biological and strongest immunomodulatory activities [134]. Fatty acids, such as 10-HDA and 3,10-DDA, have strong immunomodulatory activities exhibited commonly by the dendritic cell-associated reduction of allogeneic T-cell proliferation and IL-2 production in vitro, as well as the inhibition of the antigen-specific immune response in vivo [135]. A fatty acid 3,10-dihydroxy-decanoic acid (3,10-DDA) of RJ stimulates the maturation of monocyte-derived dendritic cells (MoDCs) by up-regulating the expression of allogeneic CD1a, CD40, CD54, and CD 86, and also boosting the allostimulatory potential in co-culture with allogeneic CD4+ T cells. The 3,10-DDA administrations to monocyte-derived dendritic cells (MoDCs) increase the production of IL-12, IL-18, and stimulate the production of IFN-ϒ in allogeneic CD4+ T cells in co-culture. Therefore, 3,10-DDA encourages maturation and Th1 polarizing potential of human MoDCs in vitro that could have an anti-viral and anti-tumor response [85]. 10-HDA has various immunomodulatory effects depending on applied concentrations. The high 10-HDA concentration could stop the function and maturation of human MoDCs and lower doses support the Th1 immune response [136].

#### 3.1.5. Anti-Aging Activity

**Figure 4:**
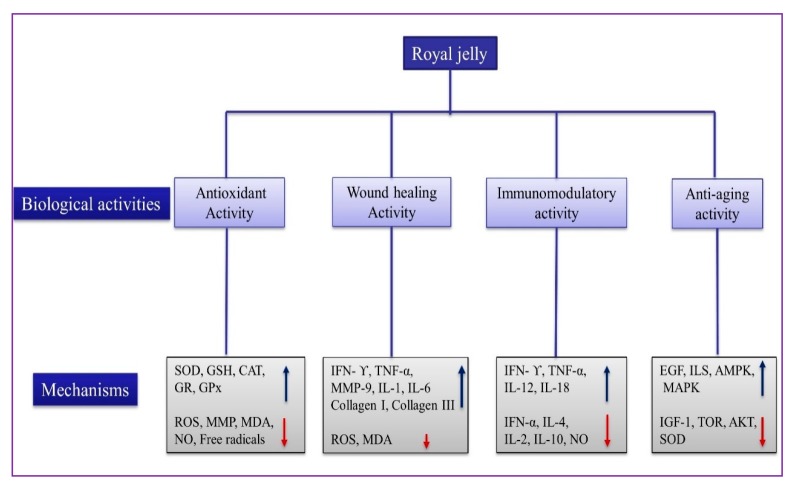
The biological activities of RJ and their mechanism. SOD (superoxide dismutase); GSH (glutathione); CAT (catalase); GR (glutathione reductase); GPx (glutathione peroxidase); ROS (reactive oxygen species); MMP (matrix metallopeptidases); MDA (malondialdehyde); NO (nitric oxide); IFN-ϒ (interferon-gamma); IL-4 (interleukin-4); TNF-α (tumor necrosis factor); IFN-α (Interferon-α); EGF (epidermal growth factor); AMPK (5′ AMP-activated protein kinase); MAPK (mitogen-activated protein kinase); IGF-1 (insulin-like growth factor-1), and TOR (target of rapamycin).

RJ is associated with an increase in the lifespan of queen honeybees as well as several other species [9], and improves the quality of life in old age rats [137]. RJ and ERJ administration have the potential to delay aging, age-related disorder, and promote longevity and stress resistance in *Caenorhabditis elegans* [138]. Furthermore, ERJ and enzyme-untreated RJ (NRJ) influence in an age focusing motor disorder in genetically heterogeneous male mice. Age-related variations affect muscle fiber size at an advanced age, muscle satellite cell markers, and catabolic genes in RJ-treated mice, thus, RJ may be useful to improve the quality of life during aging through regulating the motor functions [139]. Royalactin, a glycoprotein from RJ, extends the life span of *C. elegans* by promoting epidermal growth factor (EGF) and its receptors’ signaling [140]. MRJPs are longevity-promoting substances that increase the longevity period of *Drosophila* through promoting the anti-epidermal growth factor receptor (EGFR)-mediated signaling pathway [141]. Protein and lipid components in RJ have the potential to extend the life span in various living beings, including honeybees, crickets, silkworms, nematodes, mice, and inhibit senescence of human tissues in cell cultures via down-regulation of insulin-like growth factors and up-regulation of epidermal growth factor signaling [25]. Moreover, 10-HDA is used to increase the longevity of *C. elegans* via reduced insulin-like signaling (ILS) and increase the lifespan by dietary restriction signaling and the target of rapamycin (TOR) components in *C. elegans* [142]. The biological activities of RJ and their underlying possible mechanisms are shown in Figure 4.

### 3.2. Pharmaceutical Applications

**Figure 5:**
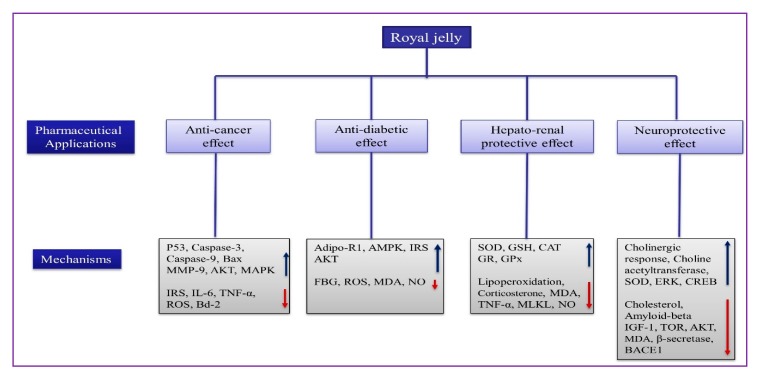
The pharmaceutical effects of RJ and their mechanism. Bax (bcl-2-like protein X); MMP-9 (matrix metallopeptidases-9); AKT (protein kinase B); MAPK (mitogen-activated protein kinase); IRS (insulin receptor substrate 1); IL-4 (interleukin-4); TNF-α (tumor necrosis factor); ROS (reactive oxygen species); AMPK (5′ AMP-activated protein kinase); SOD (superoxide dismutase); GSH (glutathione); CAT (catalase); GR(Glutathione reductase); GPx (glutathione peroxidase); MDA (malondialdehyde); NO (nitric oxide); MAKL (mixed lineage kinase domain-like); ERK (extracellular signal-regulated kinases); CREB (cAMP Response Element-Binding Protein); IGF-1 (insulin-like growth factor-1); TOR (target of rapamycin), and BACE1 (β-site amyloid precursor protein cleaving enzymes).

RJ is one of the oldest and as high potential bee medicines widely used to treat various diseases. Pharmaceutical studies elucidate that RJ has multiple activities that are attributable to their bioactive compounds, including proteins, peptides, lipids, phenolics, and flavonoid compounds. Recently, RJ has shown potential for use against cancer, diabetic, cardiovascular, and Alzheimer’s disease (AD) in modern pharmaceutical research [77,102,110]. The pharmaceutical effects of RJ (Figure 5) and its constituents in health-promotion through modulation of various biological activities are discussed below.

#### 3.2.1. Anti-Cancer Effect

RJ reveals potential anti-cancer properties as the inhibition of tumor growth and/or metastasis in the liver or lung, through the inhibition of tumor-induced angiogenesis, and/or the activation of immune function [75]. The crude RJ stops the damage of bisphenol A, which causes the enlargement of human breast cancer cells [76]. The treatment for three months with RJ exhibits better effects on decreasing the prostatic-specific antigen and ameliorates the quality of life in patients with benign prostatic hyperplasia [143]. RJ has the potential to reduce the cytotoxic effects of doxorubicin (DOX) on the prostate cancer cell line (PC3) [144]. A significant, weak, and positive correlation is found between RJ and time since diagnosis of women breast cancer and used as complementary and alternative medicine [145]. The *N*-acetylation is the main metabolic pathway which activates arylamine carcinogens that are catalyzed by *N*-acetyltransferase (NAT) and require acetyl coenzyme A. RJ affects the *N*-acetylation and inhibits the metabolism of 2-aminofluorene (2-AF) metabolites in the human liver tumor cell line and decrease the 2-AF in J5 cells in a dose-dependent manner [77]. The 10-HDA and human interferon-alpha (HuIFN-aN3) proteins have a similar activity regarding anti-tumor response and their combination decreases the level of glutathione and enhance the level of lipid peroxidation via MDA in CaCo-2 cells [78]. All lipophilic fractions from RJ share a common anti-tumoral effect against human neuroblastoma and prevent the onset, and slow down the growth of human neuroblastoma [146].

#### 3.2.2. Anti-Diabetic Effect

Many medicines are accessible on the market to control and decrease the difficulties of diabetes, but new techniques are essential to provide patients with the most therapeutic benefits and with the minimum adverse response. In a clinical study, serum glucose levels significantly decreased in healthy persons after RJ administration [80]. RJ supplementation showed remarkable decreases in serum glycosylated hemoglobin levels and fasting blood glucose (FBG) levels by increasing insulin concentration, which may help to control diabetes outcomes [79]. RJ administration enhances the total antioxidant capacity and reduces the homeostasis model assessment for insulin resistance in type-2 diabetic patients [147]. The administration (100 mg/kg) of RJ to *diabetes mellitus* rats for six weeks improves the urine parameters including uric acid, urea, albumin, creatinine, and histopathological variation of liver and kidney [81,82]. The intake of RJ possesses enviable response on serum glucose, apolipoprotein A1 (ApoA-1) concentrations, and (ApoB/ApoA-1) ratios that may decrease cardiovascular attack in people with type-2 diabetes [148]. Long-term RJ intake ameliorates hyperglycemia and partially decreases body weight in overweight/diabetic KK-Ay mice through the up-regulation of mRNA expression of adiponectic, adiponectin receptor-1, and AMP-activated protein kinase [149]. In randomized clinical trials, RJ administration (1000 mg/daily) decreases the occurrence of cardiovascular disease by attenuating the effect of fasting blood glucose level, systolic blood pressure, and interleukin-6 in patients with type-2 diabetes [150]. RJ also has the potential to reduce the irregular status of 30 mM glucose conditions in human endothelial cells under diabetic situations [151]. The splenic tissue repair in diabetic rats by RJ via increasing antioxidant enzymes and reducing glucose levels [110]. Furthermore, RJ considerably improves the serum level of triglycerides, low-density lipoprotein, very low-density lipoprotein, high-density lipoprotein, cholesterol, and ApoA-1 in diabetic patients, thereby promoting the glycemic status, oxidative stress, and lipid profile [83].

#### 3.2.3. Anti-Hypercholesterolemic Effect

RJ reduces the total lipids and cholesterol level in serum, liver of rat, rabbits, and also decreases the total serum, lipids, and cholesterol level in humans [87]. One of the early studies reported that RJ has hypocholesterolemic response to be associated with a reduction of the gene expression of squalene epoxidase and an increase of low-density lipoprotein receptor in mice [97]. The dietary RJ administration reduces the total cholesterol and low-density lipoprotein levels by decreasing the small range of very-low-density lipoprotein levels (VLDL) [86]. The treatment with RJ in rats reduces the plasma levels of triglyceride and insulin, without affecting blood glucose, total cholesterol levels, and tends to lower systolic blood pressure [152]. Oral administration of RJ (150 mg/kg) for 12 weeks could improve the lipid profile and control menopause-associated dyslipidemia in woman [88]. RJ administration continuously for three months considerably decreases the total cholesterol and LDL-c level by improving the (HDL-c) level that mitigates the chance of cardiovascular disease [90]. The first meta-analysis was conducted to determine the efficacy of RJ consumption on blood lipid parameters. The pooled analysis indicate that the RJ ingestion may effective for the development of blood lipid parameters via decreasing total cholesterol and increasing HDL-C levels in the blood concentrations [153]. MRJP-1 has a strong hypocholesterolemic effect in rats due to the interaction with bile acid that increased fecal bile acid excretion, fecal cholesterol excretion, and hepatic cholesterol catabolism [89].

#### 3.2.4. Anti-Hypertension Effect

Hypertension has become a cardiovascular risk factor that may cause heart failure, myocardial infarction, cerebral stroke, and metabolic syndrome in humans worldwide. Despite the various drugs recommended to lower hypertension it is important to achieve better solutions, and following that line various natural molecules are under investigation in drug discovery for this purpose. Oral administration of RJ reduces the systolic blood pressure, diastolic blood pressure, and enhances the NO level in SHR, both in an in vivo hypertension model and in an isolated rabbit thoracic aorta rings model. RJ causes vasorelaxation through inhibiting L-NAME (nitric oxide synthase inhibitor), indomethacin (cyclooxygenase inhibitor and atropine-M3 receptor blocker), and methylene blue (guanylate cyclase inhibitor) in isolated aortic rings. Additionally, RJ could inhibit high K^+^-induced extracellular Ca^2+^ influx and NE-induced intracellular Ca^2+^ releases in the denuded aortic ring. Furthermore, RJ could also increase NO production in cyclic guanosine monophosphate (cGMP) in isolated aortic rings. Anti-hypertensive activities of RJ are associated with NO production while muscarinic receptor agonists produced a vasodilation response by the NO/cGMP pathway and calcium channels [93]. RJ proteins have the potential to inhibit the angiotensin 1-converting enzyme’s (ACE) activity produced by the gastrointestinal enzyme hydrolysis and reduce systolic blood pressure in the spontaneously hypertensive rats (SHR) [154]. The presence of MRJP-1 protein in vascular smooth muscle cells (VSMCs) decreases cell proliferation, migration, contraction, and also reduce hypertension via influence on VSMCs [92]. However, ERJ and its peptides (Ile-Tyr, Val-Tyr, and Ile-Val-Tyr) stop ACN activities and exhibited anti-hypertensive effects after oral treatment for 28 days in the SHR. Systolic blood pressure decreases in SHR depending upon the quantity of oral administration of these peptides, which may be beneficial for improving blood pressure in people with hypertension [91].

#### 3.2.5. Anti-Inflammatory Effect

The inflammatory process is stimulated by a wide cascade of biological and chemical aspects, including cytokines, pro-inflammatory enzymes, and low molecular weight compounds (eicosanoids) or the enzymatic breakdown of tissues [155]. RJ administration successfully inhibits the production of pro-inflammatory cytokines such as IL-1, -6, and TNF-α in a dose-dependent manner without having a cytotoxic influence on macrophage in vitro [95]. RJ could be important for the improvement of quality of life in the autoimmune diseases including rheumatoid arthritis and inflammatory bowel diseases [95]. The effect of oral RJ administration has studied on acetic acid-induced colitis in the different cell lines of rats. The proliferative reaction of CD3+ and CD45+ T-cell stimulate with colitis is significantly affected when treated with RJ and no difference is found in CD5+ T-cell and CD68+ cell. Furthermore, RJ has an anti-inflammatory action and cell regeneration response on acetic acid-induced colitis rats [156]. The anti-inflammatory response of RJ could induce renal inflammation in the rats with the use of ethylene glycol. The presence of pro-inflammatory/anti-inflammatory cytokines, such as TNF-α, IL-1β, and IL-18 levels in the blood and renal tissue of rats, reflect that anti-inflammatory response of RJ due to its anti-radicals and anti-oxidative effects [94]. The dietary RJ administration improves metabolic effect and skeletal muscle functions in aged obese rats. Furthermore, RJ ameliorates the insulin resistance and muscle lipotoxicity in the aged obese rats via suppression of TNF-1 in the serum and adipose tissues due to its anti-inflammatory response [157]. RJ administration could improve the inflammatory response in microglial cells by suppressing phosphorylation of p38, an inhibitor of kappa B (IkBa), and c-jun NH2-terminal kinases (JNK), and by stopping the nucleus translocation of nuclear factor kappa B (NF-kB) and p-65 [158]. Due to anti-inflammatory properties ERJ has the potential to be developed as food to enhancing immune activities for the prevention of inflammatory disease [159]. As a unique compound in RJ, 10-HDA significantly inhibits the activities of matrix metalloproteinases (MMP-1, MMP-3), p38, and the c-Jun N-terminal kinases-activating protein-1 (JNK-AP-1) signaling pathway, which could serve as a protective tool against the therapy of rheumatoid arthritis [160]. Moreover, 10-HDA inhibits lipopolysaccharide (LPS)-induced inhibitor of kappa-B-zeta (IkB-z) and IL-6 productions, which contribute to autoimmune and inflammatory diseases [161]. The trans-10-HDA, 10-HDA, and SEA in RJ indicates the in vitro anti-inflammatory response to the release of major inflammatory–mediators, IL-10, NO, and only SEA reduced TNF-α production. However, 10-HDA indicates a stronger anti-inflammatory effect compared to the other fatty acids [162]. For the treatment of an inflammatory disorder, the natural molecule in RJ adenosine N1-oxide (ANO) is used in intravenous and oral administration of ANO decreases the lethality to lipopolysaccharide-induced endotoxin shocks [163].

#### 3.2.6. Organo-Protective Effect

##### Hepato-Renal Protective Effect

RJ is a potential alternative for the treatment of hepatic and renal dysfunctions. Dietary administration of RJ (200 mg/kg) for seven days as a hepato-protective agent could improve the severe liver damage induced by paracetamol in mice [50]. The treatment with RJ, before and after cisplatin-induced renal stress in rats, remarkably ameliorates the levels in serum of uric acid, urea nitrogen, bilirubin, and total protein, suggesting a protective response to the harmful effect of cisplatin [122]. RJ administration may be the potential preventive agent to hepatic toxicity induced by cisplatin causing histological changes in hepatic tissue through free radical scavenging, anti-oxidant properties, and anti-apoptotic stimulation [96]. RJ could be considered as beneficial to inhibit liver toxicity induced by side effects of oxymetholone (OXM) and azathioprine through reducing the activities of serum hepatic enzymes and MDA formation [164,165]. RJ has a hepato-protective effect against oxidative impairment, decreasing lipoperoxidation and corticosterone, and enhancing total antioxidant capacity in liver tissue after stress induction in rats [166]. In addition, RJ treatment ameliorates the renal ischemia/reperfusion injury in rats via reducing blood urea nitrogen, kidney MDA, leukocyte infiltration, creatinine, adhesion molecule-1 expression, glomerular diameter, the level of TNF-α, and increased the tissue ferric reducing/antioxidant power [99]. MRJP-2 could relieve hepatic necrosis against carbon tetrachloride (CCl4)-induced hepatotoxicity via inhibiting TNF-α, intercellular reactive species, and mixed lineage kinase domain-like protein (MLKL). Moreover, MRJP-2 could be a safe and reliable therapeutic approach for fighting against hepatic diseases in future human experiments [98].

##### Neuroprotective Effect

RJ plays a key role in brain cell differentiation like neuron from cultured neural progenitor cells/neural stems (NPCs/NSs), and also produces neurogenesis in the hippocampal dentate gyrus in an in vivo model [57,100]. RJ administration could stimulate neurite outgrowth from a cultured PC12 line [57]. Orally, RJ treatment improves the neural function due to the regeneration of hippocampal granule cells, which is critical for the cognition process [54], and also protects the brain from oxidative injury [167]. In humans, the intake of 3 g of RJ for six months upgrades glucose tolerance, erythropoiesis, and mental health [168]. Oral RJ treatment (100 mg/kg) reduces the apoptotic cell number in traumatic spinal cord injury in rabbits through decreasing the lipid peroxidation and increasing the endogenous enzymatic and non-enzymatic antioxidative protection system [169]. The potential effect on the central nervous system by RJ has been verified having once reduced the degree of damage and death of brain tissue, and 10-HDA contributes to helping in the generation of neurons [170]. RJ in treatments of neurological indications has proved to be effective on menopausal disorders in postmenopausal model rats. Memory impairment and depression-like behaviors in ovariectomized rats are recovered to the levels of sham-operated rats by RJ administration [171]. RJ administration has the potential to consolidate the learning, memory abilities, and longevity of honeybees [112], and the intake of RJ has the potential to ameliorate cognitive deficits [103]. RJ ingestion is effective on neurological disorders, such as Alzheimer’s disease in postmenopausal patients, via decreasing cholesterol and amyloid-beta deposition, increasing cholinergic response, estrogen level, and also improving the blood-brain barrier and autonomic nervous systems [102]. Moreover, RJ significantly improves the behavioral deficits and image structure of the brain of the cholesterol feed rabbits via decreasing body weight, level of lipid in blood, amyloid-beta, acetyl-cholinesterase, and MDA level while increasing choline acetyltransferase and SOD levels in brain tissues [101]. The purified RJ peptides (RJPs) are neuroprotective and could suppress beta-amyloid 40 and beta-amyloid 42 production by the down-regulation of β-secretase, β-site amyloid precursor protein cleaving enzymes (BACE1), and serving as a natural product to the treatment of neurodegenerative Alzheimer’s disease in aged people [101]. Furthermore, 10-HDA could inhibit the production of oligodendrocytes, astrocytes, and stimulates the differentiation of neurons from neural stem cells (NSCs) [100]. Active components, such as AMP and AMP N1-oxide, isolated from RJ are reported to induce neural differentiation and generate astrocytes of NPCs/NSs in pheochromocytoma (PC12) cell lines [55,58]. The AMP N1-oxide action is mediated by adenyl cyclase-couple adenosine receptor such as A2a [55], elevate the phosphorylation of extracellular signal-regulated kinase ½ (ERK ½) and cAMP-response element-binding protein (CREB) in neural progenitors or neural stem cells (NPCs/NSs) of the cultured PC12 line [54,57,100].

##### Other Protective Effects

RJ has the various protecting effects that could contribute to improving body functions. For instance, RJ has a protective role against radiation-induced apoptosis in human peripheral blood leukocytes through its free radical scavenging and antioxidant activities [172]. RJ indicates a genoprotective effect against DOX-induced genotoxicity in human lymphocytes and a protective mechanism probably mediated by anti-aging, anti-apoptotic, and antioxidant activities of RJ [104]. RJ administration exerted protective response against tyrosine kinase inhibitors (TKIs)-induced anorexia, fatigue, and plays a key role in sustaining the quality of life and medicine compliance in tyrosine kinase inhibitor-treated renal cell carcinoma patients [173]. RJ treatment indicates that the paclitaxel-induced histopathological and biochemical changes could be protected, and the cardioprotective effect might be correlated with the suppression of nitrosative and oxidative stress [111]. RJ exhibits a strong protective response against the cadmium exposure-induced nephrotoxicity in mice through facilitating cadmium excretion, replacing oxidant/antioxidant balance, preventing apoptosis, and inflammation [174]. 10-HDA may have the potential to protect the skin from ultraviolet (UVβ)-induced photo-aging by improving collagen production [105].

#### 3.2.7. Effect on sexual Dysfunction and Fertility

Sexual dysfunctions and fertility deficiencies are both common clinical difficulties with limited therapeutic choices. RJ is shown to improve the fertility of male and female as discussed below.

##### Estrogenic Effect

RJ has an estrogenic response in in vivo and in vitro models via the interaction with estrogen receptors followed by the alteration of gene expression and cell proliferation [175]. Orally and intramuscularly, RJ administration with exogenous progesterone ameliorates the estrous effect and pregnancy rate in Awassi ewes [176]. The oral consumption of an RJ (1000 mg/daily) capsule is effective in decreasing the severity of premenstrual syndrome (PMS) and also improves the quality of life of women during their reproductive age [177]. It also effective for improving urinary and sexual problems, and ameliorates the quality of life in postmenstrual women [178]. RJ administration could improve the in vitro fertilization capacity of Nili-Ravi buffalo bull sperm and post-thaw quality [179]. RJ treatment may induce higher levels of oocyte maturation, fertilization, and blastocyst formation by enhancing the activity of glycolysis, pentose phosphate pathway, and antioxidant enzyme activity in oocyte and cumulus cell [180]. The administration of RJ endorses follicular growth and ovarian hormones in immature female rats through its estrogenic effects on the reproductive system to improve the fertility parameters [181]. Four bioactive compounds, including 10-H2DA, 2-DEA, 10-HDA, and 24-MET of RJ, indicate an estrogen receptor-β (ERβ) response and stopped the binding activity of 17-β estradiol to ERβ in an in vivo experiment [182]. 10-HDA, 10-HDDA, and SEA present strong estrogenic effects mediating estrogen signaling by modulating the recruitment of ERα, ERβ, and co-activator to target genes [183].

##### Effect on Fertility

RJ administration significantly increases sperm active motility, luteinizing hormones, and testosterone levels in infertile men [106]. Long-term feeding of RJ increases the testosterone levels and spermatogenesis by stopping the age-associated decline in testicular function of male hamsters [108]. Co-administration of honey and RJ could be effective in the treatment of infertility due to asthenozoospermia [184]. RJ administration improves the physiological status, such as boosted testosterone level, increased ejaculation, number of sperm, sperm motility, and improved seminal plasma fructose to heat-stress rabbits, which can restrain their summer infertility [185], and sperm parameters of ram semen in the case of liquid storage [186]. Moreover, RJ could improve summer infertility and the physiological status of heat-stressed male rabbits [187]. RJ could be protective against the negative effects of flunixin meglumine-induced sperm toxicity in the male reproductive system [188]. The co-administration of RJ and cod liver oil could improve the biochemical, hormonal, and structural aspects of testicular tissue in food yellow 4 (FY4)-induced rats [107]. The co-administration could effectively improve sperm characteristics and early embryo development as well as the sperm lipid peroxidation level by acting as a promising reproductive agent in heat-stress conditions [189].

### 3.3. Side Effect of RJ Consumption

RJ causes some allergic reactions, such as contact dermatitis, anaphylaxis, and asthma, due the presence of major allergens MRJP-1 and MRJP-2 [190,191,192]. Other adverse effects also cause eczemas and skin rashes [193], respiratory stress [194], bronchospasm, hemorrhagic colitis, and even death [195,196]. The oral administration of RJ may induce an allergic reaction and some minor side effects, such as light gastrointestinal problems, atopy to serious reactions like anaphylactic shock, acute asthma, intestinal bleeding, and even death [197]. RJ and honeybee venom share common allergic substances, such as immunoglobulin E (IgE), that are responsible for the onset of occupational asthma [198]. MAJR-1 and MAJR-2 are IgE-binding allergenic proteins in RJ. Therefore, protease-treated RJ efficiently ruins the allergenic protein through significantly reducing the IgE-binding capacity in in vitro assays of the blood from RJ-sensitive patients. An in vivo human skin-prick test and histamine-release test significantly decrease the allergic response of RJ in the patient through anaphylaxis. Thus, it is valuable to prepare hypoallergenic RJ through protease treatment for its safe consumption [109,192]. Additionally, the person who has other allergies or allergic problems from bee stings, honey, or asthma, pregnant and lactating women, as well as small children should avoid RJ intake [199].

## 4. Conclusions and Future Research Directions

RJ has been used in medical products, health nutrients, cosmetics, and commercial purposes. Different biological compounds, such as protein, peptides, peptides fraction, fatty acid, and phenolic compounds isolated from RJ, lend this product various biological activities and pharmaceutical applications. Due to this, RJ is endowed with functional properties, such as antimicrobial, antioxidant, wound healing, immunomodulatory, anti-aging, anti-cancer, anti-inflammatory, anti-hypertension, anti-hyperlipidemic, estrogenic, and neurotrophic effects. Additionally, RJ could play an important role in liver and kidney protection, and improving the reproduction system. Moreover, diabetic patients provide a hypoglycemic response through decreasing lipid peroxidation levels and improve the activities of antioxidant enzymes, such as SOD, CAT, GR, and GPx. RJ could act also as a functional food and may have a protective effect on the appearance of gastrointestinal diseases. Despite a large number of bioactive compounds that have been identified, future studies are needed in the identification, extraction, and isolation of hidden bioactive compounds and their functions. The use of bioactive compounds from RJ as alternative drugs in the clinical applications are not yet implemented, thus, more evidence would help to prove the efficacy, safety, exact amount, and quality that would be required to achieve promising health benefits. Therefore, more in vivo and in vitro experiments (animal studies and clinical trials) and validation are demanded to reveal the cellular and molecular mechanisms of RJ for health benefits using cutting-edge genomics, transcriptomics, proteomics, and metabolomics.

## Figures and Tables

**Table 1:** The biological activities and pharmaceutical applications of RJ and their bioactive ingredients.

Bioactive Compounds/Experimental Models	Effects	Sources
RJ, MRJP-2, and MRJP-4 (Micro-organisms)	Antibacterial, antifungal, anti-yeast Induce damage and dysfunction in microbial cell wall and membrane	[66,67,68]
Royalisin and 10-HDA (Micro-organisms)	Antibacterial (Gram+, Gram−), antifungal Inhibit growth	[9,69,70]
Jelleine I-III, jelleine-II (pS), and jelleine-II (pT) (Micro-organisms)	Antibacterial (Gram+, Gram−) Cell degranulation, hemolysis, and increase immune defense	[39,43]
RJ, 10H∆2DA, 3,10-HDA, 11S, 10-HDA, 10-acetooxy-2-DEA, and Native jelleine-11 (Micro-organisms)	Antifungal and anti-yeast Strongly inhibit growth	[39,43,71,72,73]
Pre and post administration of RJ (Animals)	Antioxidant activity Decrease oxidative stress (MDA) and increase antioxidant properties (CAT, GPx, and SOD)	[74]
RJ (Humans)	Anti-cancer effect Inhibit the tumor-induced angiogenesis, activate immune system, metabolism of 2-AF metabolites, and stop the damage of bisphenol A	[75,76,77]
Intravenously application of 10-HDA and the HuIFN-aN3 (Animals)	Anti-cancer effect Decrease the level of glutathione and enhance the level of lipid peroxidation via MDA	[78]
RJ (Animals and humans)	Anti-diabetic effect Improve the serum level of triglycerides, lipoprotein, and cholesterol Decrease glucose level and increase insulin concentration	[79,80,81,82,83]
MRJP-3 (Animals)	Immunomodulatory effect Decrease antigenicity and inhibit IL-4, IL-2, and IFN-ϒ production	[84]
3,10-DDA (Humans)	Immunomodulatory effect Increase the production of IL-12, IL-18, and stimulate the production of IFN-ϒ	[85]
RJ (Animals and humans)	Hypocholesterolemic effect Reduced the level of triglyceride, insulin, total lipids, and cholesterol level by decreasing very low-density lipoprotein levels	[86,87,88]
RJ and MRJP-1 (Humans)	Hypocholesterolemic effect Decreased the total cholesterol and LDL-c level by improving the (HDL-c) level	[89,90]
RJ, ERJ, And MRJP-1 (Animals)	Anti- hypertension effect Reduce systolic blood pressure, diastolic blood pressure, and increase NO level	[62,91,92,93]
RJ (Animals)	Anti-inflammatory effect RJ inhibit the TNF-α, IL-1β, and, IL-18 levels in the blood due to its antiradicals and antioxidative effect	[94,95]
RJ and MRJP-2 (Animals)	Hepato-renal protective effect Reduce blood urea, MDA level, leukocyte infiltration, creatinine, adhesion molecule-1 expression, glomerular diameter, and TNF-a Increased SOD and GPx	[50,96,97,98,99]
RJ and 10-HDA Animals	Neurotrophic effects Inhibited production of oligodendrocytes, astrocytes, and stimulate neuron differentiation	[57,100]
RJ and RJPs (Animals and humans)	Neuroprotective Decrease cholesterol and amyloid-beta deposition by down-regulation of β-secretase Increase cholinergic response, estrogen level, and antioxidant capacities Improved blood-brain barrier, and autonomic nervous systems	[101,102,103]
RJ (Humans)	Genoprotective effect Increase of BCL2/BAX ratio for cell survival Enhance in hTERT/BAX for increasing age Increase in NRF2/BAX for antioxidative response	[104]
RJ and 10-HDA (Humans)	Protective effect Protect it from photo-aging by improving collagen production via up-regulation of TGF-β1 expression	[105]
RJ (Animals and humans)	Effect on fertility Increase sperm motility, luteinizing hormones, and testosterone levels	[81,106,107,108]
ERJ (Humans)	Anti-allergic Significantly reducing IgE-binding capacity of blood	[109]

Note: RJ (royal jelly); RJPs (purified royal jelly peptides); RJPH (royal jelly protein hydrolysate); MRJP-4 (ajor royal jelly protein 4); 10-HDA (10-hydroxydecanoic acid); 10H∆2DA (10-hydroxy-Delta-2-decenoic acid); 3,10-DDA (3,10-dihydroxy-decanoic acid); MDA (malondialdehyde); GPx (Glutathione peroxidase); SOD (superoxide dismutase); IFN-ϒ (interferon-gamma); IL-4 (interleukin-4); TNF-α (tumor necrosis factor); BCL2: (B-cell lymphoma 2); BAX (BCL2 associated X protein); NRF2 (nuclear factor erythroid 2 related factor 2); 2-AF (2-aminofluorene); BACE1 (β-site amyloid precursor protein cleaving enzymes), and IgE (Immunoglobulin E).

## Abbreviations

ACE	Angiotensin 1-converting enzymes
AD	Alzheimer’s disease
AF	Aminofluorene
AMP	Adenosine monophosphate
ANO	Adenosine N1-oxide
ApoA-1	Apolipoprotein A1
BACE1	β-site amyloid precursor protein cleaving enzymes
CAT	Catalase
CCl4	Carbon tetrachloride
cGMP	Cyclic guanosine monophosphate
Con-A	Concanavalin A
DecDA	1,10-decanedioic acid
DOX	Doxorubicin
EGF	Epidermal growth factor
EGFR	Anti-epidermal growth factor receptor
ERJ	Enzyme-treated RJ
FB	Fumonisin
FBG	Fasting blood glucose
FY4	Food yellow 4
GPx	Glutathione peroxidase
GR	Glutathione reductase
GSH	Glutathione
9-HDA	9-hydroxy-2-decenoic acid
10-HDA	10-hydroxy-2-decenoic acid
3-HHDA	3-hydroxydecanoic acid
10-HDDA	10-hydroxydecenoic acid
3,10-HDecDA	3,10-dihydroxydecanedioic acid
8-HOC	8-hydroxy octanoic acid
HSV-1	Herpes simplex virus type 1
HuIFN-aN3	Human interferon-alpha
IFN-ϒ	Interferon-gamma
IgE	Immunoglobulin E
IkBa	Inhibitor of kappa B
IkB-z	IkappaBzeta
IL	Interleukin
ILS	Insulin-like signaling
JNK-AP-1	C-Jun *N*-terminal kinases-activating protein-1
MDA	Malondialdehyde
24-MET	24-methylene cholesterol
MLKL	Mixed lineage kinase domain-like protein
MoDCs	Monocyte-derived dendritic cells
MRJPs	Major royal jelly proteins
NAT	*N*-acetyltransferase
NO	Nitric oxide
NPCs/NSs	Neural progenitors or neural stem cells
OXM	Oxymetholone
PC3	Prostate cancer cell line
RJ	Royal jelly
RJBs	Royal jelly bees
SEA	Sebacic acid
SHR	Spontaneously hypertensive rats
SLE	Systemic lupus erythematosus
SOD	Superoxide dismutase
TKIs	Tyrosine kinase inhibitors
VLDL	Very low-density lipoprotein levels
VSMCs	Vascular smooth muscle cells
